# Effect of precooking and polyphosphate treatment on the quality of microwave cooked catfish fillets

**DOI:** 10.1002/fsn3.465

**Published:** 2017-02-22

**Authors:** Carissa H. Li, John M. Bland, Peter J. Bechtel

**Affiliations:** ^1^ Food Processing and Sensory Quality Research UnitUSDA‐ARSSouthern Regional Research CenterNew OrleansLAUSA

**Keywords:** Catfish, microwave, polyphosphate, precooked products, texture

## Abstract

In the U.S. market place, there are many examples of precooked poultry products designed to be reheated in a microwave oven and, to a lesser extent, fish products such as tilapia. However, few U.S. catfish products are designed to be microwave cooked or reheated. The first objective of this study was to examine the properties of microwave cooked raw frozen catfish fillets and oven precooked (to 62.8°C) frozen fillets and then reheated by microwave cooking. The second objective was to evaluate changes in properties as a consequence of treatment with a commercial polyphosphate blend (Individually Quick Frozen [IQF]). The sample analysis included weight loss, proximate content, color (CIE L*a*b*), pH, mechanical texture (hardness), and lipid peroxidation (TBARS) measurements. Frozen fillets which contained polyphosphate showed <4% moisture loss after microwave cooking, relative to a 12% moisture loss for fillets without polyphosphate. A large cooking loss of ~40% was observed for precooked fillets after microwave cooking, correlated with a higher percent moisture loss (11% and 13% for fillets with and without polyphosphate, respectively) to comparable samples that were not precooked. For both types of fillets, an increased amount of yellow color was observed for precooked fillets after microwave cooking, relative to comparable fillets that were not precooked. Fillet hardness determined by peak force revealed an overall harder texture (~1.1–1.8 times) for fillets without polyphosphate than fillets with polyphosphate. This study will be used to develop precooked catfish products that can be reheated in a microwave oven.

## 1 Introduction

Precooking is a processing technology that applies heat to food and its products at relatively low heat flow for certain amount of time or until a safe minimum internal temperature is observed. The FDA requires fish and shell fish to reach a minimum of 62.8°C (145°F) for 15 s to be considered precooked and ready to eat (FDA, 1999a). However, comminuted fish and foods containing comminuted fish shall be cooked to heat all parts of the food to 68°C (154°F) for 15 s (FDA, 1999b) and stuffed fish or stuffing containing fish shall be cooked to heat all parts of the food to 74°C (165°F) for 15 s (FDA, 1999c). It is one of the critical steps in processing perishable foods for extended storage time such as frozen meat or seafood products (Lee, Yang, & Kim, 2002). From the safety standpoint, the mild thermal treatment preserves the quality of the original foods while ceasing the enzymatic and bacterial activities which are potentially harmful to human body, therefore providing enhanced safety and mitigating processor risk especially in the event of unexpected thermal processing deviations executed by consumers (Belongia et al., 1991; Patsias, Chouliara, Badeka, Savvaidis, & Kontominas, 2006; Smith et al., 2008; Parra, Kim, Shapiro, Gravani, & Bradley, 2014; Gupta, Gandotra, Koul, Gupta, & Parihar, 2015). As precooked foods are generally safe and require minimal cooking prior to consumption, there are numerous ways to cook or reheat precooked products. Examples included baking or steaming in an oven, boiling in water, roasting on a grill, or deep fat frying in oil, with each method offering different taste or texture. Cooking in a microwave oven is another commonly used method to prepare precooked products. Unlike the traditional cooking methods by which temperature increases slowly with a heat source, foods become hot in a microwave oven due to the energy release in the form of heat as a result of vibration of water molecules. With the advantages of short cooking time, less preparation in advance, as well as quick and easy clean‐up after uses, microwaving has become one of the most popular ways for cooking lunch boxes or other forms of precooked products (Cross, Fung, & Decareau, 1982).

In addition to safety, another predominant factor that affects consumers' readiness in buying precooked products is the quality of food. As the majority of precooked products in the market are designed with extended shelf life, precooked foods are generally stored frozen; therefore, changes in quality as a consequence of dehydration, protein denaturation, or tissue cell breakage are commonly observed (Matlock, Terrell, Savell, Rhee, & Dutson, 1984; Sikorski & Sunpan, 1992; Chang, Chang, Shiau, & Pan, 1998; Arannilewa, Salawu, Sorungbe, & Ola‐Salawu, 2005). The excess thermal treatment history also potentially decreases the quality of precooked products by reducing moisture content or hardening texture (Panea, Sañudo, Olleta, & Civit, 2008; Półtorak et al., 2015). Therefore, finding ways to determine or preserve the quality of precooked products is essential. Polyphosphate treatment, most commonly consisting of a blend of sodium tripolyphosphate and hexametaphosphate, is a widely employed technique in the meat and seafood industry to preserve quality of food. It has been used to effectively enhance muscle juiciness and reduce drip loss and cooking loss during cooking, freezing, and frozen storage, where myofibrillar proteins readily denature and lose the majority of their water binding capacity (Dyer, Brockerhoff, Hoyle, & Fraser, 1964; Morey, Satterlee, & Brown, 1982; Woyewoda & Bligh, 1986; Sheard, Nute, Richardson, Perry, & Taylor, 1999). When properly used, polyphosphate treatment gives no flavor while retarding oxidative deterioration of muscle by chelating heavy metal ions (Lampila, 1993). Besides retaining natural muscle moisture and preventing lipid oxidation, the ability of increasing thermal stability of proteins and imparting cryoprotection is also beneficial to preserving quality of food (Etemadian, Shabanpour, Sadeghi Mahoonak, Shabani, & Alami, 2011).

In the U.S. market place, there are many examples of precooked poultry products designed to be reheated in a microwave oven and, to a lesser extent, fish products such as tilapia. However, precooked catfish products designed to be microwave cooked remain in a relatively small portion. The present study investigated the quality of raw frozen catfish fillets, microwave cooked, or oven precooked fillets reheated by microwave cooking. As precise control on cooking time and microwave power plays an important role in determining the quality of the final product, preliminary cooking experiments were performed to explore the optimum microwave conditions. Finally, changes in chemical and mechanical properties as a consequence of treatment with a commercial polyphosphate blend were also evaluated. This work is expected to provide practical information to the catfish industry in the preparation of future frozen catfish products that are designed to be cooked or reheated in a microwave oven.

## 2 Materials and Methods

### 2.1 Sample preparation

Both fresh and IQF (containing a commercially injected polyphosphate blend) catfish samples were purchased from a commercial catfish processor in Mississippi. Fillets were placed in plastic bags and stored frozen at −20°C until cooked or analyzed. They were separated into four treatment groups: raw (R), precooked (P), precooked plus microwave cooked (P + M), and microwave cooked (M). For each group, two randomly selected fillets (5–7 oz) were trimmed and cut into three pieces (two front, two middle, and two end pieces) each weighing approximately 50 g.

### 2.2 Precooking method

Thermocouples (company name) were inserted into the center of samples in groups P and P + M, which were precooked on a pan in a 121°C (250°F) convection oven to an internal temperature of 60°C (140°F). After reaching an internal temperature of 60°C (140°F), samples were removed from oven and placed on a rack where the internal temperature continued to increase. Initial studies found that after samples were removed from the oven, the fillet internal temperature increased to an average final temperature of 62.8°C (145°F) for ~50 g fillet pieces. After the precooking step, samples were then stored frozen at −20°C until cooked by microwave or analyzed.

### 2.3 Cooking or reheating method

Both fresh and IQF samples that required a final microwave cooking process (groups P + M and M) were prepared by microwaving the precooked frozen or raw frozen fillets on medium (870 W) for 2.5 min (3.5 min for IQF samples), which resulted in internal temperature of 93°C (200°F). Once cooked, they were individually placed in ziploc bags and stored frozen at −20°C until analyzed.

### 2.4 Cooking loss calculation

Cooking loss was calculated as percent weight loss from before to after cooking. For samples in group P + M, weight loss for both precooking and microwave cooking processes was recorded, and cooking loss was calculated as the total percent weight loss before precooking and after microwave cooking.

### 2.5 Proximate analysis

Proximate analysis was performed on ground samples. Moisture content was determined gravimetrically, in triplicate, after drying the sample in a 103°C oven for 24 hr. The sample was then collected and used to analyze ash and protein content. Ash content was obtained gravimetrically by heating the dried sample in a muffle furnace (Thermal Scientific Lindberg Blue M) at 550°C for 5 hr. Protein analysis was performed in triplicate using a FP628 nitrogen analyzer (LECO Co., St. Joseph, MI), and protein content was determined by multiplying the results by 6.25. EDTA was employed as the calibration standard. Pseudo lipid content was obtained by subtracting percent moisture, ash, and protein from 100. All data presented in this article were based on wet weight unless otherwise specified.

### 2.6 Colorimetric measurements

CIE L* (lightness), a* (redness), b* (yellowness) color values were obtained on both the surface and vertical cross‐section (internal) of the fillet using a CR‐410 Chroma meter (Konica Minolta, Ramsey, NJ) with 50 mm aperture size, 2° observer, illuminant C, and 0° viewing angle. Before measuring the samples, the instrument was calibrated using a standard white CR A44 Minolta calibration plate. Four areas were measured on each sample piece.

### 2.7 Texture analysis

Mechanical texture of the cooked sample pieces was measured on a TA.XT*plus* Texture Analyzer (Stable Micro Systems Ltd., Godalming, UK) with a 30‐kg load cell using a TA‐18 (half inch diameter ball) probe. Hardness was determined as the peak positive force with exponent software during a single cycle compression to 50% of the sample height (thickness) at 1 mm/s pretest speed, 1 mm/s test speed, and 3 mm/s posttest speed. Samples in plastic bags were thawed in a cold water bath to room temperature and texture was measured at two areas per sample piece before vertically cutting the sample pieces into half for internal colorimetric measurements.

### 2.8 pH measurement

Samples for pH measurements were prepared by homogenizing 2 g of sample (wet weight) in 20 ml Nanopure water (resistivity 18.2 MΩ cm) for 30 s. After samples were homogenized, 20 ml of Nanopure water was used to rinse‐off residuals left on the probe of homogenizer and added to sample solution. The pH values were measured using a ɸ350 pH meter (Beckman Instruments, Inc., Fullerton, CA) with a glass electrode.

### 2.9 Thiobarbituric acid reactive substances (TBARS) determination

A 5 g (wet weight) sample was mixed with 10 ml of 10% (w/v) trichloroacetic acid (TCA), and the mixture was homogenized for 90 s. After removing the precipitate, 2 ml of supernatant was mixed with 2 ml of TBA reagent (20.8 mmol/L). The mixture was then heated in a dry bath for 20 min at 94°C, giving a transparent pink solution. Amount of malondialdehyde (MDA) was obtained by reading the absorbance at 532 nm on a Lambda 35 UV/Vis Spectrometer (PerkinElmer, Inc., Beaconsfield, UK). Results were expressed as μg MDA per gram of wet sample.

### 2.10 Statistical analysis

All data presented in the article were statistically analyzed by one‐way analysis of variance (ANOVA), *p *<* *.05. Comparison was performed between treatments as well as between fresh and IQF fillets. A Tukey's test was used for all pairwise multiple comparison. For analyses that failed normality or equal variance tests, a Kruskal–Wallis ANOVA on ranks was used, with a Dunn's method used for all pairwise multiple comparison. A Mann–Whitney rank sum test was used to compare fresh and IQF results.

## 3 Results and discussion

### 3.1 Effect of fillet size on cooking time

**Figure 1:**
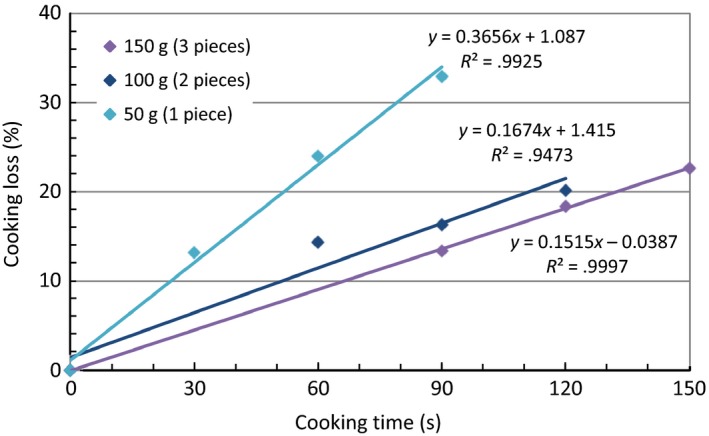
Correlation between cooking loss and cooking time of three different fillet sizes (fresh samples). The linear regression lines and equations were shown in the plot

A preliminary microwave cooking experiment was conducted to study the time required to microwave cook different weights of fresh catfish fillets: 50 g, 100 g, and 150 g (one, two, and three fillet pieces) to an internal temperature of 93°C (200°F). The higher end temperature of 93°C (200°F), considering 62.8°C (145°F) is the minimum internal temperature required for cooked fish as suggested by Food and Drug Administration (FDA, 1999a), was chosen to minimize temperature deviation caused by variation in fillet size or thickness, after microwaving for the desired length of time. All samples were cooked on high in a covered Corningware dish with a 1.45‐kW microwave oven. For a fillet piece weighing approximately 50 g, the internal temperature increased from 16°C (61°F) to 54°C (129°F) to 97°C (207°F) after microwave cooking for 0.5 min, 1 min, and 1.5 min, respectively. A 0.5‐ to 1‐min increment in cooking time was required for each additional fillet piece to cook to similar internal temperatures. When cooking loss was plotted as a function of cooking time, a linear correlation was observed (Figure 1). A smaller number of fillets cooked faster, resulting in a significantly greater cooking loss.

### 3.2 Effect of microwave power on cooking time and moisture content

**Figure 2:**
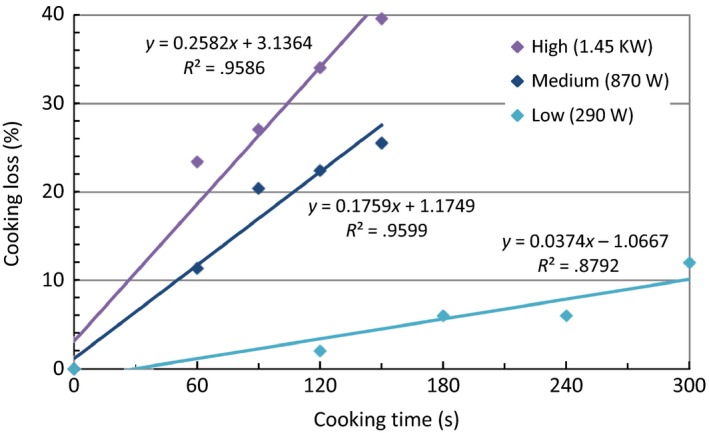
Correlation between cooking loss and cooking time of fresh catfish fillets cooked at three different microwave power levels. The linear regression lines and equations were shown in the plot

To evaluate the effect of microwave power on cooking, fresh catfish fillet pieces each weighing approximately 50 g were cooked in a microwave oven at three different power levels, low (290 W), medium (870 W), and high (1.45 kW). Correlation between cooking time and cooking loss was shown in Figure 2. The cooking was relatively nonuniform, with the coldest part being the bottom of fillet, until an internal temperature of ~93°C (200°F) was observed (2–2.5 min on medium and 1.5 min on high). At comparable cooking conditions (internal temperature and cooking loss), fillets cooked on high (1.45 kW) lost significantly more moisture (14%) than fillets cooked on medium (870 W), which had a 9.6% moisture loss. Based on preliminary results, cooking on medium (870 W) for 2.5 min was determined as the optimum condition for cooking a ~50 g catfish fillet using a microwave oven.

### 3.3 Cooking loss

**Figure 3:**
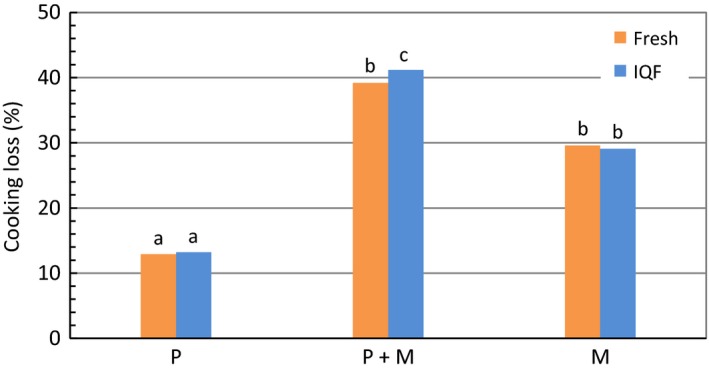
Comparison of cooking loss for fresh and IQF fillets between various treatments. Bars marked with the same letter designate no significant difference (*p *˃ .05) between treatments

**Figure 4:**
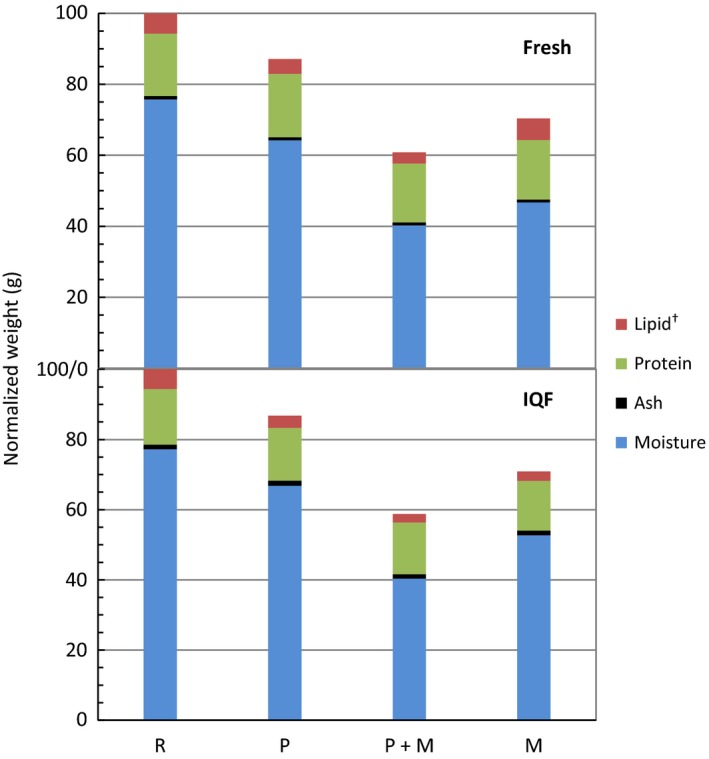
Percent weight after cooking treatments and proximate compositions for both fresh and IQF fillets between various treatments. Values were normalized to 100 g for raw (or uncooked) fillets. Weights for the cooked fillets were subsequently calculated by subtracting the cooking loss

Figure 3 shows the cooking loss for fresh and IQF samples of various treatments. An increase in cooking loss in the microwave treatments was in agreement with one report that showed cooking in a microwave oven results in a greater cooking loss relative to products cooked by conventional methods (Kylen, McGrath, Hallmark, & Van Duyne, 1964). The P + M treatments showed the greatest cooking loss, with its value equaling the sum of the P and M treatments, and being significantly different from the M treatment in IQF samples. The larger cooking loss for microwave treatments (P + M or M) was accompanied by a higher percent moisture loss (Figure 4). A similar loss was seen whether the fillets contained polyphosphate (IQF) or not. There was no significant difference between fresh and IQF samples for all treatments.

### 3.4 Proximate analysis

The results of proximate analysis for both fresh and IQF catfish samples are shown in Table 1. The percent moisture of the fresh samples with P + M treatment was not significantly different from the M treatment; however, for the IQF samples there was a significant difference between the P + M and M treatments, with a P + M moisture loss of 11% compared to 3.8% for M (Figure 4 and Table 1). Consistent with previously reported results, the moisture retention properties were significantly greater for fillets with polyphosphate treatment (IQF), relative to fresh fillets (Regenstein, Jauregui, & Baker, 1984; Etemadian et al., 2011). The effect was particularly perceptible for raw frozen fillets after microwave cooking (M), with <4% moisture loss relative to 12% for fresh samples with similar treatment.

**Table 1:** Proximate composition of fresh and IQF catfish fillets of various treatments

	R	P	P + M	M
Fresh				
Moisture (%)	75.73^a^	73.70^a^	66.18^b^	66.38^b^
Ash (%)	0.97^a^	1.04^a^	1.42^b^	1.14^ab^
Protein (%)	17.60^a^	20.53^a^	27.27^b^	23.77^b^
Lipid^a^ (%)	5.70^ab^	4.74^b^	5.14^ab^	8.71^a^
IQF				
Moisture (%)	77.23^a*^	76.99^ab^*	68.71^c^*	74.33^b^*
Ash (%)	1.31^a^*	1.69^b^*	2.12^c^*	1.86^bc^*
Protein (%)	15.81^a*^	17.29^a^*	24.95^c^*	20.06^b^*
Lipid^a^ (%)	5.65^a^	4.04^a^	4.21^a^	3.76^a^*

Values for IQF fillets having an asterisk (*) indicate a significant difference between fresh and IQF for the corresponding treatment. Values with the same superscript letter, within a row, are not significantly different (*p *˃ .05).

a Pseudo lipid content obtained by subtracting percent moisture, ash, and protein from 100.

A relatively larger standard deviation was found in percent ash within treatments for IQF fillets, possibly reflecting a nonuniform polyphosphate distribution both between fillets and between positions (front, middle, or end) within a fillet. The more fillets were cooked (P + M ˃ M ˃ P ˃ R), the greater the percent ash was for both types of fillets (fresh or IQF), despite the differences not being significant among most treatments on a dry weight basis. Comparing fresh to IQF samples, all IQF samples showed a significantly higher (0.3%–0.7%) percent ash than the comparable fresh samples as expected. These results were consistent with the results obtained from phosphate analysis, in which an average of 0.3% and 0.6% polyphosphate was measured for raw and cooked IQF samples, respectively. The higher ash and polyphosphate numbers observed in cooked IQF fillets indicated negligible polyphosphate loss after cooking while percent moisture became lower.

A similar trend of increasing percent protein (P + M ˃ M ˃ P ˃ R) was observed for both types of samples (fresh or IQF), with fresh fillets having significantly greater (1.8%–3.7%) percent protein than IQF fillets. Within a type of fillets (fresh or IQF), significant differences were found between raw (R) or precooked (P) and microwave cooked (P + M or M) samples.

Percent lipid was calculated to be 4%–6% (except for fresh M), consistent with the reported values for catfish fillets (Mustafa & Medeiros, 1985). The microwave cooked (M) fresh samples had a higher lipid value of 8.7%, possibly due to the high percent moisture loss relative to the comparable IQF samples. All fresh samples contained greater percent lipid than IQF samples, with significant difference found in group M.

### 3.5 Color analysis

**Figure 5:**
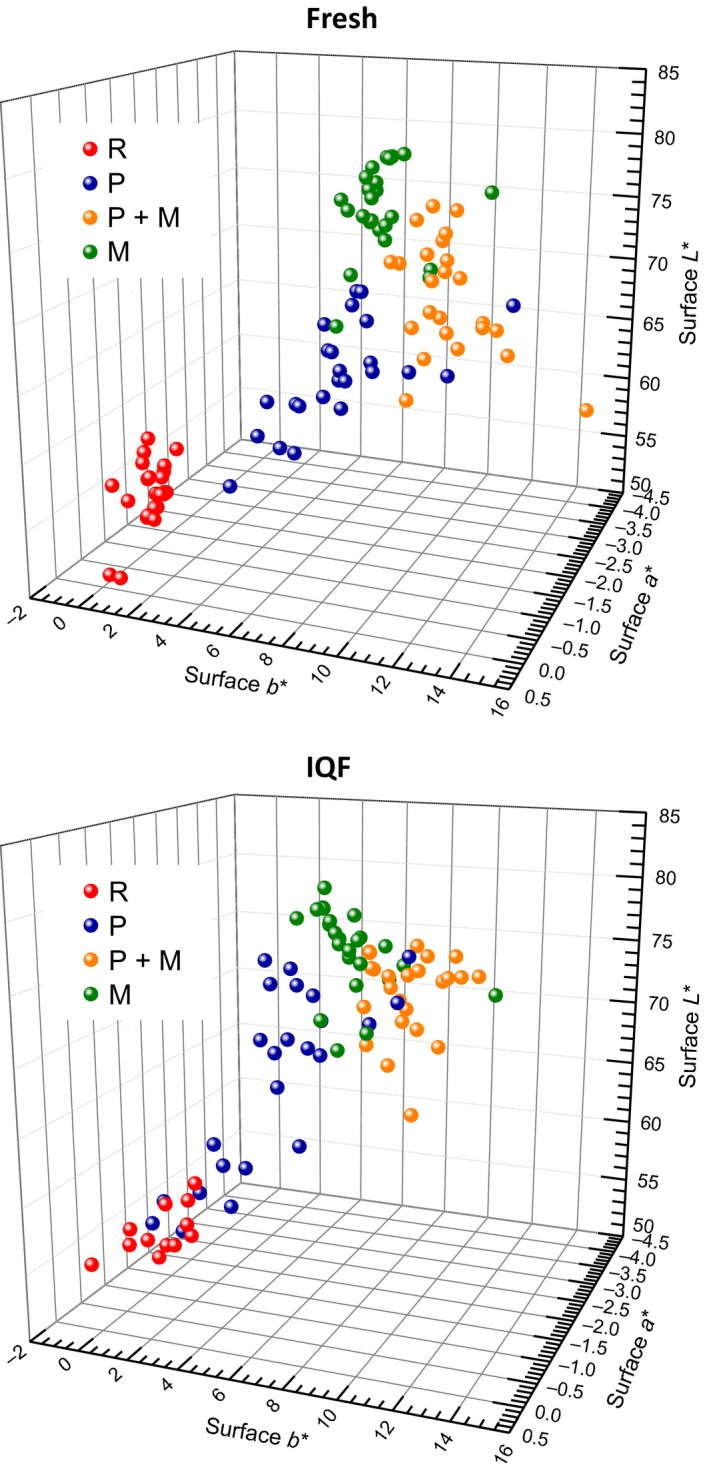
Comparison of surface L*a*b* color values for both fresh and IQF catfish fillets between various treatments. Positive a* = degree of redness, positive b* = degree of yellowness, L* = lightness. Different treatments were noted with different colors of balls

When color of the surface of fresh or IQF fillets was determined, groupings were observed showing separation between treatments along the L*(C) (lightness), a*(C) (redness), and b*(C) (yellowness) axis (Figure 5). The microwave cooked (M) samples showed the greatest lightness and least amount of redness for both types of fillets, followed by groups P + M, P, and R. The amount of yellowness reflected the history of cooking, with the double cooked (P + M) samples showing significantly greater yellow color than microwave cooked (M) samples. All cooked fresh samples (P, P + M, or M) had a significantly greater amount of red and yellow color than the comparable IQF samples.

**Figure 6:**
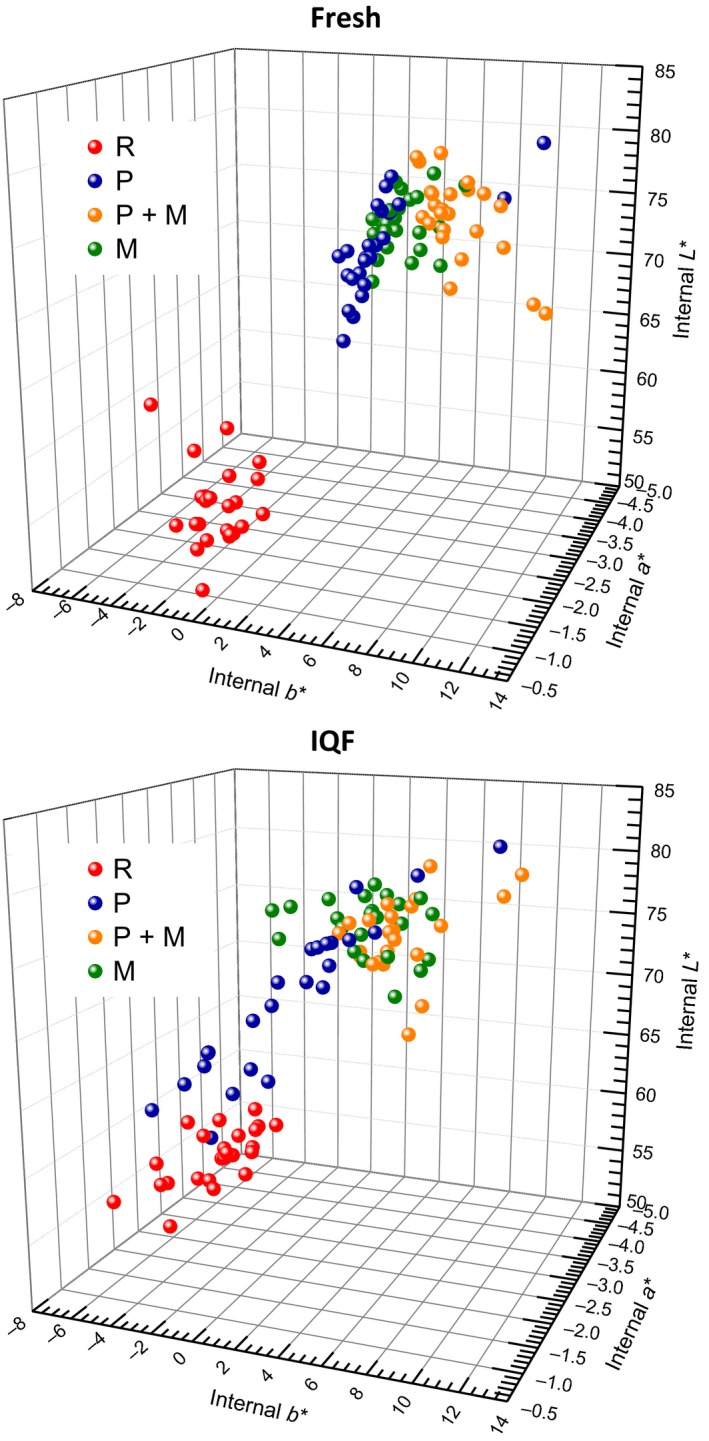
Comparison of internal (vertical cross‐section surface) L*a*b* color values for both fresh and IQF catfish fillets between various treatments. Positive a* = degree of redness, positive b* = degree of yellowness, L* = lightness. Different treatments were noted with different colors of balls

Although differences in lightness between different cooked treatments were distinguishable when color was determined on the surface of fillets, all cooked samples (P, P + M, or M), for both fresh and IQF fillets, had similar lightness on the cross‐section when fillets were vertically cut into half (Figure 6). Similar to surface color, all cooked fresh samples (P, P + M, or M) showed a greater amount of internal lightness, redness, and yellowness than the comparable IQF samples.

### 3.6 pH value

Samples which were cooked in a microwave oven (P + M or M) had a slightly higher pH value than samples that were not (R or P) (Table**** 2). However, the differences were not considered significant between treatments or types of cooked fillets, except group M IQF samples had a significantly higher pH value than fresh samples with similar treatment.

**Table 2:** The pH values of fresh and IQF catfish fillets of various treatments

pH	R	P	P + M	M
Fresh	6.94	6.89	7.17	7.02
IQF	6.95	6.92	7.07	7.21*

Value for IQF fillets having an asterisk (*) indicates a significant difference between fresh and IQF for the corresponding treatment.

### 3.7 Texture analysis

**Figure 7:**
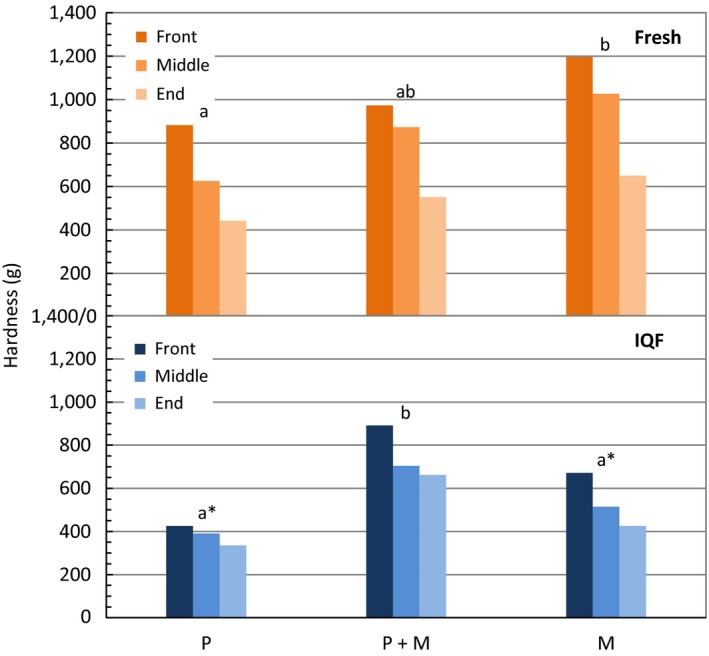
Mechanical texture analysis (hardness) of fresh and IQF fillets of various treatments. Results were plotted separately by position of fillet (front, middle, or end). When position of fillet was considered as a random variable, bars marked with the same letter on top, within the same type of fillet, are not significantly different (*p *˃ .05). Significant differences between fresh and IQF for the corresponding treatment were noted with an asterisk on the graph of IQF samples

Correlation between position of fillet and mechanical texture for both cooked fresh and cooked IQF fillets was shown in Figure 7. A general trend of decreasing hardness was observed from front to middle to end pieces (decreasing fillet thickness). After cooking in a microwave oven, precooked (P + M) fresh samples did not show significant difference in texture relative to non‐precooked (M) fresh samples, whereas a significantly harder texture (1.4 times) was measured for precooked (P + M) IQF samples when compared to raw frozen microwave cooked samples (M). As previously reported, polyphosphate treatment increases juiciness and tenderness of meat and seafood products due to the weakened muscle structure (Klose, Campbell, & Hanson, 1963; Griffiths & Wilkinson, 1978; Sheard et al., 1999). All fresh fillets had a harder texture than IQF fillets, with significant differences found in groups P and M (1.7 and 1.8 times, respectively).

### 3.8 TBARS level

**Figure 8:**
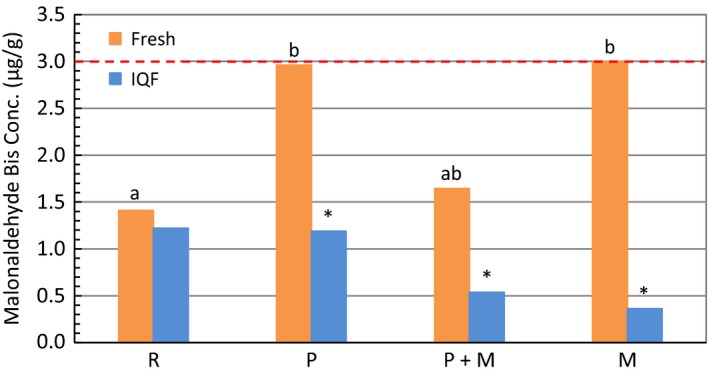
Comparison of TBARS values for fresh and IQF fillets between various treatments. The threshold of MDA concentration that is considered good for consumption was marked with a red dotted line. Bars marked with the same letter designate no significant difference (*p *˃ .05) between treatments. Significant differences between fresh and IQF for the corresponding treatment were noted with an asterisk above the bar of IQF samples

To evaluate the extent of lipid oxidation as a consequence of various treatments, malonaldehyde (MDA) concentration, one of the main end products of lipid oxidation, was measured by performing TBARS assay. Figure 8 compares the MDA concentration of both fresh and IQF fillets of various treatments. Although similar MDA concentrations were detected for raw fresh and raw IQF fillets, IQF fillets showed significantly lower TBARS level than fresh fillets after being cooked (P, P + M, or M) (Lampila, 1993). It is worth noting that these results are consistent with the percent lipid of the samples as shown in Figure 4. One report showed MDA could be lost during cooking by dissolution or formation of adducts with proteins (Weber, Bochi, Ribeiro, Victório, & Emanuelli, 2008). A lower TBARS level was measured when a greater percent lipid loss was observed after cooking (raw fresh ≈ raw IQF; lipid loss of fresh <IQF for groups P, P + M, or M). No significant difference was found between different cooked treatments (P, P + M, or M) within fillet type.

## 4 Conclusion

The microwave cooked products from raw or precooked fresh catfish fillets did not show a significant proximate composition, MDA concentration, pH value, or texture difference. However, after cooking in a microwave oven, precooked IQF fillets had significantly lower percent moisture, higher percent protein, and harder texture relative to samples that were not precooked. The longer fillets were thermally treated for, the greater the amount of yellow color was. Therefore, a significantly greater yellowness was observed on the surface of microwave cooked samples which were precooked, for both fresh and IQF fillets. The precooked fresh pieces also showed a significantly lower lightness on the surface and a greater yellowness on the cross‐section surface. Comparison of fresh to IQF fillets showed IQF fillets to have significantly higher percent moisture and ash, lower percent protein, softer texture (P or M), lower MDA concentration, and less amount of surface and internal red and yellow color, relative to the comparable fresh fillets. The smaller sample size of approximately 50 g fillet pieces, high end temperature of 93°C (200°F), or limited number of fillet used for each treatment in the research may give rise to slight variations in product quality when practiced by industry. Ongoing research includes a storage study of precooked fillet pieces which may be of benefit to the development of safe precooked catfish products that can be reheated in a microwave oven.

## Conflict of Interest

None declared.

## References

[fsn3465-bib-0001] Arannilewa, S. T. , Salawu, S. O. , Sorungbe, A. A. , & Ola‐Salawu, B. B. (2005). Effect of frozen period on the chemical, microbiological and sensory quality of frozen tilapia fish (*Sarotherodun galiaenus*). African Journal of Biotechnology, 4, 852–855. doi:10.5897/AJB2005.000‐3171 10.1177/026010600601800210 16859181

[fsn3465-bib-0002] Belongia, E. A. , MacDonald, K. L. , Parham, G. L. , White, K. E. , Korlath, J. A. , … Osterholm, M. T. (1991). An outbreak of *Escherichia coli* 0157:H7 colitis associated with consumption of precooked meat patties. Journal of Infectious Diseases, 164, 338–343. doi:10.1093/infdis/164.2.338 1856483 10.1093/infdis/164.2.338

[fsn3465-bib-0003] Chang, K. L. B. , Chang, J. , Shiau, C. Y. , & Pan, B. S. (1998). Biochemical, microbiological, and sensory changes of sea bass (*Lateolabrax japonicus*) under partial freezing and refrigerated storage. Journal of Agriculture and Food Chemistry, 46, 682–686. doi:10.1021/jf970622c 10.1021/jf970622c 10554299

[fsn3465-bib-0004] Cross, G. A. , Fung, D. Y. C. , & Decareau, R. V. (1982). The effect of microwaves on nutrient value of foods. Critical reviews in food science and nutrition, 16, 355–381. doi:10.1080/10408398209527340 7047080 10.1080/10408398209527340

[fsn3465-bib-0005] Dyer, W. J. , Brockerhoff, H. , Hoyle, R. J. , & Fraser, D. I. (1964). Polyphosphate treatment of frozen cod. I. Protein extractability and lipid hydrolysis. Journal of the Fisheries Board of Canada, 21, 101–106. doi:10.1139/f64‐008

[fsn3465-bib-0006] Etemadian, Y. , Shabanpour, B. , Sadeghi Mahoonak, A. R. , Shabani, A. , & Alami, M. (2011). Cryoprotective effects of polyphosphates on *Rutilus frisii kutum* fillets during ice storage. Food Chemistry, 129, 1544–1551. doi:10.1016/j.foodchem.2011.06.005

[fsn3465-bib-0007] FDA . (1999a) Destruction of organisms of public health concern: Cooking (raw fish) Section 3–401.11(A)(1), 1995. Food Code, Food and Drug Administration. (Pp. 53). Washington, DC: United States Public Health Service.

[fsn3465-bib-0008] FDA . (1999b). Destruction of organisms of public health concern: Cooking (comminuted fish) Section 3–401.11(A)(2), 1995. Food Code, Food and Drug Administration. (Pp. 54). Washington, DC: United States Public Health Service.

[fsn3465-bib-0009] FDA . (1999c). Destruction of organisms of public health concern: Cooking (stuffed fish) Section 3‐401.11(A)(4), 1995. Food Code, Food and Drug Administration. (Pp. 54). Washington, DC: United States Public Health Service.

[fsn3465-bib-0010] Griffiths, N. M. , & Wilkinson, C. C. L. (1978). The effects on broiler chicken of polyphosphate injection during commercial processing. II. Sensory assessment by consumers and an experienced panel. International Journal of Food Science and Technology, 13, 541–549. doi:10.1111/j.1365‐2621.1978.tb00835.x

[fsn3465-bib-0011] Gupta, V. , Gandotra, R. , Koul, M. , Gupta, S. , & Parihar, D. S. (2015). Quality evaluation and shelf life assessment of raw and value added fish product (fish cutlet) of *Wallago attu* during frozen storage conditions (‐12°C). Int J Fish Aquat Stud, 2, 243–247.

[fsn3465-bib-0012] Klose, A. A. , Campbell, A. A. , & Hanson, H. L. (1963). Influence of polyphosphates in chilling water on quality of poultry meat. Poultry Science, 42, 743–749. doi:10.3382/ps.0420743

[fsn3465-bib-0013] Kylen, A. M. , McGrath, B. H. , Hallmark, E. L. , & Van Duyne, F. O. (1964). Microwave and conventional cooking of meat. Journal of the American Dietetic Association, 39, 139–145. 14180628

[fsn3465-bib-0014] Lampila, L. E. (1993). Functions and uses of phosphates in the seafood industry. Journal of Aquatic Food Product Technology, 1, 29–41. doi:10.1300/J030v01n03_04

[fsn3465-bib-0015] Lee, Y. C. , Yang, H. S. , & Kim, D. H. (2002). Shelf‐life determination of precooked frozen pork meat patties at various temperatures. Journal of Food Processing and Preservation, 26, 165–177. doi:10.1111/j.1745‐4549.2002.tb00478.x

[fsn3465-bib-0016] Matlock, R. G. , Terrell, R. N. , Savell, J. W. , Rhee, K. S. , & Dutson, T. R. (1984). Factors affecting properties of precooked‐frozen pork sausage patties made with various NaCl/phosphate combinations. Journal of Food Science, 49, 1372–1375. doi:10.1111/j.1365‐2621.1984.tb14993.x

[fsn3465-bib-0017] Morey, K. S. , Satterlee, L. D. , & Brown, W. D. (1982). Protein quality of fish in modified atmospheres as predicted by the C‐PER assay. Journal of Food Science, 47, 1399–1400. doi:10.1111/j.1365‐2621.1982.tb04947.x

[fsn3465-bib-0018] Mustafa, F. A. , & Medeiros, D. M. (1985). Proximate composition, mineral content, and fatty acids of catfish (*Ictalurus punctatus, Rafinesque*) for different seasons and cooking methods. Journal of Food Science, 50, 585–588. doi:10.1111/j.1365‐2621.1985.tb13749.x

[fsn3465-bib-0019] Panea, B. , Sañudo, C. , Olleta, J. L. , & Civit, D. (2008). Effect of ageing method, ageing period, cooking method and sample thickness on beef textural characteristics. Spanish Journal of Agricultural Research, 6, 25–32. doi:10.5424/sjar/2008061‐291

[fsn3465-bib-0020] Parra, P. A. , Kim, H. , Shapiro, M. A. , Gravani, R. B. , & Bradley, S. D. (2014). Home food safety knowledge, risk perception, and practices among Mexican‐Americans. Food Control, 37, 115–125. doi:10.1016/j.foodcont.2013.08.016

[fsn3465-bib-0021] Patsias, A. , Chouliara, I. , Badeka, A. , Savvaidis, I. N. , & Kontominas, M. G. (2006). Shelf‐life of a chilled precooked chicken product stored in air and under modified atmospheres: microbiological, chemical, sensory attributes. Food Microbiology, 23, 423–429. doi:10.1016/j.fm.2005.08.004 16943033 10.1016/j.fm.2005.08.004

[fsn3465-bib-0022] Półtorak, A. , Wyrwisz, J. , Moczkowska, M. , Marcinkowska‐Lesiak, M. , Stelmasiak, A. , … Sun, D. W. (2015). Microwave vs. convection heating of bovine Gluteus Medius muscle: impact on selected physical properties of final product and cooking yield. International Journal of Food Science and Technology, 50, 958–965. doi:10.1111/ijfs.12729

[fsn3465-bib-0023] Regenstein, J. M. , Jauregui, C. A. , & Baker, R. C. (1984). The effect of pH, polyphosphates and different salts on water retention properties of ground trout muscle. Journal of Food Biochemistry, 8, 123–131. doi:10.1111/j.1745‐4514.1984.tb00320.x

[fsn3465-bib-0024] Sheard, P. R. , Nute, G. R. , Richardson, R. I. , Perry, A. , & Taylor, A. A. (1999). Injection of water and polyphosphate into pork to improve juiciness and tenderness after cooking. Meat Science, 51, 371–376. doi:10.1016/S0309‐1740(98)00136‐3 22062033 10.1016/s0309-1740(98)00136-3

[fsn3465-bib-0025] Sikorski, Z. F. , & Sunpan, B. (1992). Preservation of seafood quality In Shahidi F., & Botta J. R. (Eds.), Seafoods: chemistry, Processing Technology and Quality. London, UK: Blackie Academic & Press.

[fsn3465-bib-0026] Smith, K. E. , Medus, C. , Meyer, S. D. , Boxrud, D. J. , Leano, F. , … Danila, R. N. (2008). Outbreaks of Salmonellosis in Minnesota (1998 through 2006) associated with frozen, microwaveable, breaded, stuffed chicken products. Journal of Food Protection, 71, 2153–2160. 18939771 10.4315/0362-028x-71.10.2153

[fsn3465-bib-0027] Weber, J. , Bochi, V. C. , Ribeiro, C. P. , Victório, A. M. , & Emanuelli, T. (2008). Effect of different cooking methods on the oxidation, proximate and fatty acid composition of silver catfish (*Rhamdia quelen*) fillets. Food Chemistry, 106, 140–146. doi:10.1016/j.foodchem.2007.05.052

[fsn3465-bib-0028] Woyewoda, A. D. , & Bligh, E. G. (1986). Effect of phosphate blends on stability of cod fillets in frozen storage. Journal of Food Science, 51, 932–935. doi:10.1111/j.1365‐2621.1986.tb11202.x

